# Diagnostic and Prognostic Impact of Progesterone Receptor Immunohistochemistry: A Study Evaluating More Than 16,000 Tumors

**DOI:** 10.1155/2022/6412148

**Published:** 2022-08-08

**Authors:** Florian Viehweger, Lisa-Marie Tinger, David Dum, Natalia Gorbokon, Anne Menz, Ria Uhlig, Franziska Büscheck, Andreas M. Luebke, Claudia Hube-Magg, Andrea Hinsch, Doris Höflmayer, Christoph Fraune, Patrick Lebok, Sören Weidemann, Maximilian Lennartz, Frank Jacobsen, Till S. Clauditz, Rainer Krech, Till Krech, Andreas H. Marx, Ronald Simon, Eike Burandt, Stefan Steurer, Guido Sauter, Sarah Minner, Christian Bernreuther

**Affiliations:** ^1^Institute of Pathology, University Medical Center Hamburg-Eppendorf, Hamburg, Germany; ^2^Institute of Pathology, Clinical Center Osnabrueck, Osnabrueck, Germany; ^3^Department of Pathology, Academic Hospital Fuerth, Fuerth, Germany

## Abstract

Progesterone receptor (PR) is a member of the nuclear/steroid hormone receptor family of ligand-dependent transcription factors. It plays an important role in reproduction and mammary gland development and has various tissue-specific effects in nonreproductive organs. In diagnostic pathology, positive PR immunostaining is used to support a diagnosis of breast or gynecologic origin in a tumor. In this study, the expression of PR was analyzed by immunohistochemistry in 18,176 (interpretable: 16,445) samples from 147 different tumor types and subtypes in a tissue microarray format. PR immunostaining was detected in 57.4% of breast tumors, 28.6% of other gynecological tumors, and 1.8% of nongynecological and nonmammary tumors. Among the group of nongynecological and nonmammary tumors, particularly high rates of PR positivity were seen in neuroendocrine tumors (54.3%) and neuroendocrine carcinomas (35.7%) of the pancreas. A comparison with clinico-pathological parameters showed that reduced PR immunostaining was significantly associated with adverse histopathological and clinical features in breast carcinoma, endometrioid endometrial carcinoma, and pancreatic neuroendocrine tumors. In summary, our analysis of 147 different tumor types for PR immunostaining provides a ranking list of tumor entities according to their prevalence of PR positivity, helps to better understand the diagnostic utility of PR, and highlights the distinct PR positivity among neuroendocrine neoplasms of pancreatic origin.

## 1. Introduction

Progesterone receptor (PR) is a member of the nuclear/steroid hormone receptor family of ligand-dependent transcription factors. PR mediates the physiological effects of progesterone which plays an important role in the establishment and maintenance of pregnancy, hence the hormone's name, which comes from the Latin *pro gestationem.* In addition to its effects in reproduction and mammary gland development [1], PR is involved in the regulation of various genes, affects cellular proliferation and differentiation in various nonreproductive tissues, exerts a neurosteroid activity in the central nervous system (reviewed in [2]), inhibits smooth muscle contractile activity in the gastrointestinal tract [3], and plays a role in development and maturation of the lung [4].

In diagnostic pathology, immunohistochemical detection of PR supports the diagnosis of a carcinoma of breast or gynecologic origin if cancers of unknown primary (CUP) are being evaluated [5]. However, many studies have shown that nonbreast and nongynecological tumors can also express estrogen and/or progesterone receptor. Data on PR immunostaining in the literature typically lacks associations with patient age, gender, or stage in cancer [6–14] and is overall highly variable. For example, the reported range of PR positivity ranges from 0 to 76% in colorectal cancer [6–8], from 0 to 52% in adenocarcinoma of the gall bladder [9, 10, 15, 16], from 0 to 85% in prostate cancer [11, 17, 18], from 0 to 63% in non-small-cell lung cancer [12, 19, 20], from 38.7 to 75.8% in papillary thyroid carcinoma [13, 14, 21–24], and from 15.2 to 100% in angiomyolipoma of the kidney [25–27]. These conflicting data are likely to be caused by the use of different antibodies, staining protocols, and interpretation criteria in these studies.

To better understand the diagnostic impact of PR immunohistochemistry, a comprehensive and highly standardized study analyzing a large number of tumors, especially from nongynecological and nonbreast tissues, is needed. Therefore, PR expression was successfully analyzed in more than 16,000 tumor tissue samples from 147 different tumor types and subtypes as well as 76 different nonneoplastic tissue types by immunohistochemistry in a tissue microarray (TMA) format in this study.

## 2. Material and Methods

### 2.1. Tissue Microarrays (TMAs)

The normal tissue TMA was composed of 8 samples from 8 different donors for each of 76 different normal tissue types (608 samples on one slide). The tumor TMAs contained a total of 18,176 primary tumors from 147 tumor types and subtypes. Detailed histopathological data on grade, pT, and pN status (HER2 status for breast cancer) were available from 2,139 breast cancers, 259 endometrial cancers, 192 neuroendocrine neoplasms, and 524 ovarian tumors. Clinical follow-up data were available from 877 patients with breast cancer. In these patients, the median follow-up time was 43 (range 1-88) months. The composition of both normal and tumor TMAs is described in detail in the results section. All samples were from the archives of the Institutes of Pathology, University Hospital of Hamburg, Germany; the Institute of Pathology, Clinical Center Osnabrueck, Germany; and Department of Pathology, Academic Hospital Fuerth, Germany. Tissues were fixed in 4% buffered formalin and then embedded in paraffin. One tissue spot (diameter: 0.6 mm) was transmitted from a tumor containing donor block in an empty recipient paraffin block. The use of archived remnants of diagnostic tissues for manufacturing of TMAs and their analysis for research purposes as well as patient data analysis has been approved by local laws (HmbKHG, §12) and by the local ethics committee (Ethics commission Hamburg, WF-049/09). All work has been carried out in compliance with the Helsinki Declaration.

### 2.2. Immunohistochemistry (IHC)

Freshly prepared TMA sections were immunostained in one day in one experiment. Slides were deparaffinized with xylol, rehydrated through a graded alcohol series, and exposed to heat-induced antigen retrieval for 5 minutes in an autoclave at 121°C in pH 7.8 buffer. Endogenous peroxidase activity was blocked with Dako Peroxidase Blocking Solution™ (Agilent, CA, USA; #52023) for 10 minutes. Primary antibody specific against PR (rabbit recombinant, MSVA-570R, #3332-570R; MS Validated Antibodies GmbH, Hamburg, Germany) was applied at 37°C for 60 minutes at a dilution of 1 : 50 (final concentration: 4 *μ*g/ml). Bound antibody was then visualized using the EnVision Kit™ (Agilent, CA, USA; #K5007) according to the manufacturer's directions. The sections were counterstained with haemalaun. For the purpose of antibody validation, immunohistochemical staining of the normal tissue TMA was performed with a different antiprogesterone antibody (mouse monoclonal, PgR636, Agilent, CA, USA; # IR068) on the DAKO autostainer system. Only nuclear staining was scored. For normal tissues, the staining intensity of positive cells was semiquantitively recorded (+, ++, +++). For tumor tissues, the percentage of PR positive tumor cells was estimated, and the staining intensity was semiquantitatively recorded (0, 1+, 2+, 3+). For statistical analyses, the staining results were categorized into four groups as follows: negative: no staining at all, weak staining: staining intensity of 1+ in ≤70% or staining intensity of 2+ in ≤30% of tumor cells, moderate staining: staining intensity of 1+ in >70%, staining intensity of 2+ in >30% but in ≤70% or staining intensity of 3+ in ≤30% of tumor cells, and strong staining: staining intensity of 2+ in >70% or staining intensity of 3+ in >30% of tumor cells.

### 2.3. Statistics

Statistical calculations were performed with JMP 14 software (SAS Institute Inc., NC, USA). Contingency tables and the chi^2^-test were performed to search for associations between PR and tumor phenotype. Survival curves were calculated according to Kaplan-Meier. The log-rank test was applied to detect significant differences between groups. A *p* value of ≤0.05 was defined as significant.

## 3. Results

### 3.1. Technical Issues

An interpretable result was found in 16,445 (90.5%) tumors. Noninterpretable samples were due to lack of unequivocal tumor cells or loss of the tissue spot during technical procedures for one or both of the markers. A sufficient number of samples of each normal tissue type was evaluable.

### 3.2. Progesterone Receptor Immunostaining in Normal Tissues

**Figure 1:**
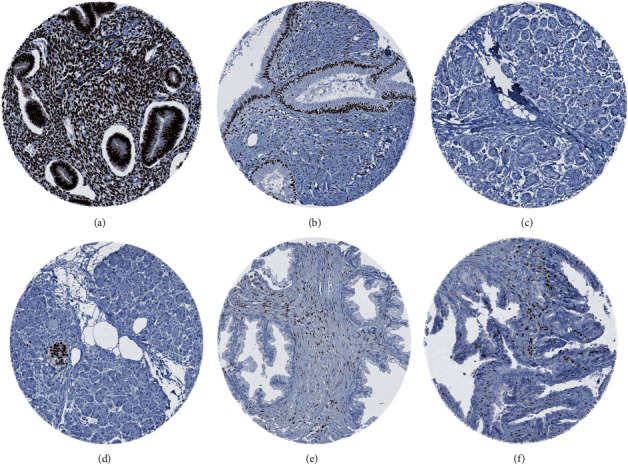
PR immunostaining in normal tissues. Positive PR immunostaining in (a) stromal cells and epithelial cells in proliferative endometrium, (b) stromal cells and epithelial cell in endocervix, (c) luminal cells of breast epithelium, (d) islets of Langerhans in pancreas, (e) stromal cells of the prostate, and (f) stromal cells of seminal vesicle.

In normal tissues, PR was expressed in various organs of the female reproductive organs, such as ovarian stroma, corpus luteum of the ovary, epithelial and stromal cells of the fallopian tube, stromal cells and basal cell layer of the squamous epithelium of the ectocervix, stromal and epithelial cells of the endocervix, stromal and epithelial cells of the endometrium, and decidual cells. In the female breast, some epithelial cells showed a moderate to strong staining. A positive immunostaining was also observed in islets of Langerhans of the pancreas, in a subset of epithelial cells of the adenohypophysis, a subset of adrenocortical cells, in a small number of epithelial cells of the submandibular gland, in subsets of glomerular, tubular and stromal cells of the kidney, in epithelial cells of the cauda epididymis, and a fraction of smooth muscle cells of the ileum, esophagus, and aorta. In some organs, only stromal cells showed a positive immunostaining. This included the prostate gland, the seminal vesicle, and the urinary bladder. PR staining was completely absent in skeletal muscle, heart muscle, fat, skin (including hair follicle and sebaceous glands), oral mucosa of the lip, oral cavity, surface epithelium of the tonsil, and transitional mucosa of the anal canal, squamous epithelium of the esophagus, urothelium of the renal pelvis and urinary bladder, corpus spongiosum of the penis, placental trophoblastic cells, mucosa of the stomach, duodenum, ileum, appendix, colon, rectum and gall bladder, liver, parotid gland, sublingual gland, Brunner gland of the duodenum, testis, respiratory epithelium and glands of bronchi and sinus paranasales, lung, thyroid and parathyroid gland, spleen, lymph node, thymus, cerebellum, and cerebrum. Images of PR staining in normal tissues are shown in Figure 1. By using the antibody PgR636, all positive stainings described above were confirmed. An additional staining of occasional mast cells, intracellular mucin within goblet cells in the tubular gut, and colloid of the thyroid gland was only seen by this antibody and was considered a tolerable antibody-specific cross-reactivity (Supplementary Figure 1).

### 3.3. Progesterone Receptor Immunostaining in Neoplastic Tissues

**Figure 2:**
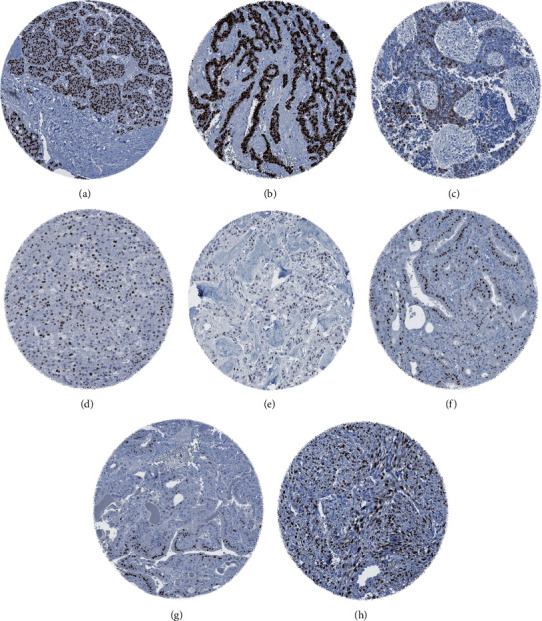
Moderate to strong PR immunostaining in “nonmammary” and “nongynecological” tumors. (a) Neuroendocrine tumor of the pancreas. (b) Neuroendocrine carcinoma of the pancreas. (c) Small cell carcinoma of the lung. (d) Leydig cell tumor of the testis. (e) Medullary thyroid carcinoma. (f) Follicular thyroid carcinoma. (g) Papillary thyroid carcinoma. (h) Angiomyolipoma.

A PR immunostaining was found in 1,856 (11.3%) of 16,445 cases (573 weak, 333 moderate, 950 strong; Table 1). 55 of 147 (37.4%) different tumor entities included at least one PR-positive case and 31 (21%) entities contained at least one tumor with strong PR staining. PR immunostaining was detected in 57.4% of breast tumors, 28.6% of other gynecological tumors, and 1.8% of nongynecological and nonmammary tumors. A ranking of tumor categories according to the rate of PR positivity is given in Table 2. Particularly, high rates of PR positivity were seen in neuroendocrine tumors (54.3%) and neuroendocrine carcinomas (35.7%) of the pancreas. The group of nonbreast and nongynecological tumors expressing PR in at least 10% of cases included also Leydig cell tumor of the testis (36.7%), medullary thyroid carcinoma (20.4%), small cell neuroendocrine carcinoma of the prostate (16.7%), small cell carcinoma of the lung (12.5%), angiomyolipoma (12.5%), adrenal cortical carcinoma (11.5%), follicular thyroid carcinoma (11.3%), and papillary thyroid carcinoma (10.6%). Images of progesterone receptor staining in “nonmammary” and “nongynecological” tumors are shown in Figure 2.

### 3.4. Progesterone Receptor Immunostaining, Tumor Phenotype, and Prognosis

Reduced PR immunostaining was significantly associated with adverse histopathological and clinical features in breast carcinoma, endometroid endometrial carcinoma, and pancreatic neuroendocrine tumors (Table 3). In breast carcinomas of no special type, reduced PR immunostaining was linked to advanced tumor stage (*p* < 0.0001), lymph node metastasis (*p* < 0.0001), high tumor grade (*p* < 0.0001), distant metastasis (*p* < 0.0001), positive HER2 status (*p* < 0.0001), and shorter overall survival (negative vs. any positivity, HR 1.8, 95% CI 1.3-2.5, *p* = 0.0127; Supplementary Figure 2). In endometroid endometrial carcinoma, low PR immunostaining was linked to lymph node metastasis (*p* = 0.0327). In 49 pancreatic neuroendocrine tumors, low PR immunostaining was linked to lymph node metastasis (*p* = 0.0345). PR staining was unrelated to histopathological features in 343 serous ovarian carcinomas. Within nonmammary, nongynecological, and nonprostate tumors, PR positivity was more common in tumors from female (3.2% of 3,085) than from male patients (1.6% of 4,752; *p* < 0.0001).

## 4. Discussion

Our successful analysis of more than 16,000 tumors revealed PR expression in 57.4% of breast tumors, 28.6% of other gynecological tumors, and 1.8% of nongynecological and nonmammary tumors.

Given the large size of our study, particular emphasis was placed on the validation of our reagents and protocols. The International Working Group for Antibody Validation (IWGAV) has proposed that antibody validation for immunohistochemistry on formalin fixed tissues should include either a comparison of the findings obtained by two independent antibodies or a comparison with expression data obtained by another independent method [28–30]. Both methods were applied in this project. A comparison of our IHC data with RNA data provided from three independent publicly available databases (Human Protein Atlas (HPA) RNA-seq tissue dataset [31], FANTOM5 project [32, 33], and Genotype-Tissue Expression (GTEx) project [34]) revealed IHC positivity in all tissues with unequivocal RNA expression such as the organs of the female genital tract, prostate, seminal vesicle, epididymis, and the pituitary gland. RNA expression had previously not been recorded for several tissues with a positive PR immunostaining such as the aortic wall, pancreatic islet cells, kidney, duodenum, adrenal gland, stroma cells of urinary bladder and pyelon mucosa, smooth muscle cells of gastrointestinal tract, or salivary glands. These tissues had previously either not been analyzed on the RNA level (aortic wall, Brunner glands of the duodenum) or the PR positive cells constitute such small fractions of their respective organs total number of cells that their PR RNAs may not have occurred at detectable quantities. True PR expression in all these cell types is supported by identical stainings obtained by the antibody PgR636 (Supplementary Figure 1). Additional positivity obtained by PgR636 in goblet cells of the gut and of thyroidal colloid was considered an antibody cross-reactivity specific to PgR636 because these tissues remained unstained by MSVA-570R.

The PR immunostaining results in breast and other gynecological tumors were in the range of most previous studies which is another confirmation of our experimental approach. The slightly lower PR positivity rate of breast tumors in our study (57.4%) compared to the 60-70% positivity rate described in previous studies (reviewed in [35]) may reflect a TMA effect. TMAs generally result in slightly lower positivity rates than seen in large section analysis. In a highly standardized study comparing PR immunostaining between TMAs and traditional sections in more than 500 breast cancers, Torhorst et al. [36] had found a PR positivity of 41-53% in multiple TMAs and 60% PR positivity in large sections. Although progesterone receptors are widely expressed in ovarian cancers, their distribution varies significantly by histology. Particularly, sex cord stromal tumors showed high PR positivity (50-81%), which fits well with previous studies [37–39]. PR positivity was found in 62% of endometroid but only in 32% of high grade serous ovarian carcinomas. This is in line with earlier studies describing 41-67% PR positivity in endometroid [40–43] but only 25-50% PR positivity in high-grade serous carcinomas of the ovary [43, 44]. In the uterus, endometrioid carcinomas (67%) also showed a much higher rate of PR positivity than serous carcinomas (21%). Consistent with these data, earlier reports have described PR positivity in 62.3-81.3% of endometroid [45–47] but only in 20-46% of serous carcinomas of the endometrium [48, 49]. As expected from previous studies (reviewed in [35, 50]) an absent or low expression of PR in breast and endometrium cancer was linked to unfavorable patient outcome. This observation seemingly reflects a loss of PR expression during cellular dedifferentiation as part of tumor progression.

Positive PR immunostaining was found in 239 tumors from 30 different categories in nonbreast and nongynecological tumors. In this group, a particularly high rate of PR positivity was observed in sex cord stromal tumors of the testis and in several neuroendocrine neoplasms. Among neuroendocrine tumors, there was a noticeable accumulation of positive cases among tumors originating from the pancreas. PR positivity was found in 54% of neuroendocrine tumors and in 36% of neuroendocrine carcinomas of the pancreas which is consistent with earlier studies describing PR positivity in 58-82% of pancreatic neuroendocrine tumors [51–53]. Given that only 0-6% of intestinal and none of the neuroendocrine tumors of the lung showed PR immunostaining, immunohistochemical PR analysis appears to represent a relevant diagnostic tool to determine the origin of metastases from neuroendocrine tumors. In concordance with our results, PR immunostaining has thus been proposed in the differential diagnosis between metastasis of small bowel neuroendocrine tumor and pancreatic neuroendocrine tumor [54]. The high rate of PR positive neuroendocrine tumors of the pancreas corresponds to the strong nuclear PR immunostaining in islets of Langerhans in normal pancreatic tissue [55, 56]. It is therefore not surprising that reduced PR staining, potentially a sign of dedifferentiation, was associated with the presence of lymph node metastasis in our pancreatic neuroendocrine tumors. Viale et al. also found reduced PR positivity associated with presence of metastases [53]. Another rare tumor entity of the pancreas, solid pseudopapillary neoplasm (which was not analyzed in this study), also was shown to express PR and therefore could come into differential diagnosis when evaluating a PR positive pancreatic tumor [51].

Other neuroendocrine neoplasms that showed PR expression in a significant fraction of cases predominantly included poorly differentiated small-cell neuroendocrine carcinomas from various sites of origin and medullary carcinoma of the thyroid. It is of note that various other tumors of the thyroid gland also showed PR immunostaining in 7-12% of cases. Other investigators have reported even higher rates of PR positivity in 39-76% of papillary thyroid carcinoma [13, 14, 21–23] and 17% of follicular thyroid carcinoma [57]. A dependency of PR in the pathogenesis of at least some thyroid cancers could explain why thyroid cancer is more than twice as common in women compared to men [58] and is the second most common type of cancer in pregnancy [59]. Bertoni et al. have demonstrated a direct effect of progesterone on thyroid cells, upregulating genes involved in thyroid function and growth [60]. Furthermore, patients receiving mifepristone, a PR blocker, had a decrease in thyroid hormone levels [61]. Interestingly, among 7,657 nonmammary and nongynecological tumors, significantly, more PR-positive tumors were seen in women (3.2%) than in men (1.6%), although there are no great quantitative differences in the progesterone serum levels between women and men outside the luteal phase [62].

In summary, our analysis of 147 different tumor types for PR immunostaining provides a ranking list of tumor entities according to their prevalence of PR positivity. Given the highly discordant literature data, such a ranking order would have been difficult to extract from the existing literature (summarized in Supplementary Figure 3). These data help to better understand the diagnostic utility of PR IHC. The distinction of neuroendocrine neoplasms derived from the pancreas appears to represent a particularly strong and poorly known application of PR IHC.

## Figures and Tables

**Table 1:** PR immunostaining in tumors.

	Tumor entity	On TMA (*n*)	PR immunostaining				
			Analyzable (*n*)	Negative (%)	Weak (%)	Moderate (%)	Strong (%)
Tumors of the skin (*n* = 410)	Pilomatrixoma	35	34	100.0	0.0	0.0	0.0
	Basal cell carcinoma	88	82	100.0	0.0	0.0	0.0
	Benign nevus	29	29	100.0	0.0	0.0	0.0
	Squamous cell carcinoma of the skin	90	90	100.0	0.0	0.0	0.0
	Malignant melanoma	46	46	100.0	0.0	0.0	0.0
	Malignant melanoma lymph node metastasis	86	84	100.0	0.0	0.0	0.0
	Merkel cell carcinoma	46	45	100.0	0.0	0.0	0.0

Tumors of the head and neck (*n* = 1,188)	Squamous cell carcinoma of the larynx	60	54	100.0	0.0	0.0	0.0
	Squamous cell carcinoma of the pharynx	60	59	100.0	0.0	0.0	0.0
	Oral squamous cell carcinoma (floor of the mouth)	80	80	100.0	0.0	0.0	0.0
	Warthin tumor of the parotid gland	55	53	100.0	0.0	0.0	0.0
	Adenocarcinoma, NOS (papillary cystadenocarcinoma)	14	11	100.0	0.0	0.0	0.0
	Salivary duct carcinoma	15	13	100.0	0.0	0.0	0.0
	Acinic cell carcinoma of the salivary gland	181	135	100.0	0.0	0.0	0.0
	Adenocarcinoma NOS of the salivary gland	109	81	97.5	0.0	1.2	1.2
	Adenoid cystic carcinoma of the salivary gland	180	126	100.0	0.0	0.0	0.0
	Basal cell adenocarcinoma of the salivary gland	25	21	100.0	0.0	0.0	0.0
	Basal cell adenoma of the salivary gland	86	66	100.0	0.0	0.0	0.0
	Epithelial-myoepithelial carcinoma of the salivary gland	53	52	100.0	0.0	0.0	0.0
	Mucoepidermoid carcinoma of the salivary gland	343	327	99.7	0.0	0.3	0.0
	Myoepithelial carcinoma of the salivary gland	21	17	94.1	0.0	5.9	0.0
	Myoepithelioma of the salivary gland	11	10	100.0	0.0	0.0	0.0
	Oncocytic carcinoma of the salivary gland	12	8	100.0	0.0	0.0	0.0
	Polymorphous adenocarcinoma, low grade, of the salivary gland	41	34	100.0	0.0	0.0	0.0
	Pleomorphic adenoma of the salivary gland	53	41	100.0	0.0	0.0	0.0

Tumors of the lung, pleura, and thymus (*n* = 382)	Adenocarcinoma of the lung	196	191	100.0	0.0	0.0	0.0
	Squamous cell carcinoma of the lung	80	75	100.0	0.0	0.0	0.0
	Small cell carcinoma of the lung	16	16	87.5	6.3	6.3	0.0
	Mesothelioma, epitheloid	39	30	100.0	0.0	0.0	0.0
	Mesothelioma, other types	76	70	100.0	0.0	0.0	0.0

Tumors of the female genital tract (*n* = 1,534)	Squamous cell carcinoma of the vagina	78	74	100.0	0.0	0.0	0.0
	Squamous cell carcinoma of the vulva	130	123	100.0	0.0	0.0	0.0
	Squamous cell carcinoma of the cervix	129	126	99.2	0.8	0.0	0.0
	Adenocarcinoma of the cervix	21	21	90.5	4.8	4.8	0.0
	Endometrioid endometrial carcinoma	236	197	33.5	21.8	13.7	31.0
	Endometrial serous carcinoma	82	68	79.4	14.7	2.9	2.9
	Carcinosarcoma of the uterus	48	41	87.8	4.9	0.0	7.3
	Endometrial carcinoma, high grade, G3	13	12	83.3	8.3	0.0	8.3
	Endometrial clear cell carcinoma	8	6	100.0	0.0	0.0	0.0
	Endometrioid carcinoma of the ovary	110	92	38.0	18.5	14.1	29.3
	Serous carcinoma of the ovary	559	520	67.9	22.1	4.2	5.8
	Mucinous carcinoma of the ovary	96	77	94.8	0.0	0.0	5.2
	Clear cell carcinoma of the ovary	50	45	88.9	6.7	2.2	2.2
	Carcinosarcoma of the ovary	47	44	65.9	22.7	6.8	4.5
	Granulosa cell tumor of the ovary	37	37	18.9	29.7	29.7	21.6
	Leydig cell tumor of the ovary	4	4	50.0	50.0	0.0	0.0
	Sertoli cell tumor of the ovary	1	1	100.0	0.0	0.0	0.0
	Sertoli Leydig cell tumor of the ovary	3	3	33.3	66.7	0.0	0.0
	Steroid cell tumor of the ovary	3	3	100.0	0.0	0.0	0.0
	Brenner tumor	41	40	95.0	0.0	0.0	5.0

Tumors of the breast (*n* = 2,051)	Invasive breast carcinoma of no special type	1764	1605	42.9	10.7	10.7	35.8
	Lobular carcinoma of the breast	363	302	43.0	9.9	9.6	37.4
	Medullary carcinoma of the breast	34	33	87.9	3.0	3.0	6.1
	Tubular carcinoma of the breast	29	23	17.4	8.7	8.7	65.2
	Mucinous carcinoma of the breast	65	51	23.5	7.8	7.8	60.8
	Phyllodes tumor of the breast	50	37	27.0	0.0	18.9	54.1

Tumors of the digestive system (*n* = 3,911)	Adenomatous polyp, low-grade dysplasia	50	50	100.0	0.0	0.0	0.0
	Adenomatous polyp, high-grade dysplasia	50	50	100.0	0.0	0.0	0.0
	Adenocarcinoma of the colon	2482	2146	99.9	0.0	0.0	0.0
	Gastric adenocarcinoma, diffuse type	176	150	100.0	0.0	0.0	0.0
	Gastric adenocarcinoma, intestinal type	174	157	100.0	0.0	0.0	0.0
	Gastric adenocarcinoma, mixed type	62	49	100.0	0.0	0.0	0.0
	Adenocarcinoma of the esophagus	83	83	100.0	0.0	0.0	0.0
	Squamous cell carcinoma of the esophagus	75	75	100.0	0.0	0.0	0.0
	Squamous cell carcinoma of the anal canal	89	88	100.0	0.0	0.0	0.0
	Cholangiocarcinoma	50	50	100.0	0.0	0.0	0.0
	Gallbladder adenocarcinoma	31	31	100.0	0.0	0.0	0.0
	Gallbladder klatskin tumor	41	39	100.0	0.0	0.0	0.0
	Hepatocellular carcinoma	300	299	100.0	0.0	0.0	0.0
	Ductal adenocarcinoma of the pancreas	612	505	97.8	1.0	0.6	0.6
	Pancreatic/ampullary adenocarcinoma	89	75	100.0	0.0	0.0	0.0
	Acinar cell carcinoma of the pancreas	16	15	100.0	0.0	0.0	0.0
	Gastrointestinal stromal tumor (GIST)	50	49	100.0	0.0	0.0	0.0

Tumors of the urinary system (*n* = 3,181)	Noninvasive papillary urothelial carcinoma, pTa G2 low grade	177	170	100.0	0.0	0.0	0.0
	Noninvasive papillary urothelial carcinoma, pTa G2 high grade	141	135	100.0	0.0	0.0	0.0
	Noninvasive papillary urothelial carcinoma, pTa G3	219	195	100.0	0.0	0.0	0.0
	Urothelial carcinoma, pT2-4 G3	735	636	99.7	0.0	0.0	0.3
	Squamous cell carcinoma of the bladder	22	22	100.0	0.0	0.0	0.0
	Small cell neuroendocrine carcinoma of the bladder	23	23	95.7	4.3	0.0	0.0
	Sarcomatoid urothelial carcinoma	25	23	100.0	0.0	0.0	0.0
	Urothelial carcinoma of the kidney pelvis	62	61	98.4	1.6	0.0	0.0
	Clear cell renal cell carcinoma	1287	1179	99.9	0.1	0.0	0.0
	Papillary renal cell carcinoma	368	329	99.7	0.3	0.0	0.0
	Clear cell (tubulo) papillary renal cell carcinoma	26	24	95.8	0.0	4.2	0.0
	Chromophobe renal cell carcinoma	170	153	95.4	3.3	0.0	1.3
	Oncocytoma	257	231	93.1	6.5	0.4	0.0

Tumors of the male genital organs (*n* = 1,350)	Adenocarcinoma of the prostate, Gleason 3 + 3	83	83	100.0	0.0	0.0	0.0
	Adenocarcinoma of the prostate, Gleason 4 + 4	80	80	100.0	0.0	0.0	0.0
	Adenocarcinoma of the prostate, Gleason 5 + 5	85	85	100.0	0.0	0.0	0.0
	Adenocarcinoma of the prostate (recurrence)	258	257	99.6	0.4	0.0	0.0
	Small cell neuroendocrine carcinoma of the prostate	19	18	83.3	16.7	0.0	0.0
	Seminoma	621	586	100.0	0.0	0.0	0.0
	Embryonal carcinoma of the testis	50	45	100.0	0.0	0.0	0.0
	Leydig cell tumor of the testis	30	30	63.3	33.3	3.3	0.0
	Sertoli cell tumor of the testis	2	1	0.0	100.0	0.0	0.0
	Sex cord stromal tumor of the testis	1	1	0.0	0.0	100.0	0.0
	Spermatocytic tumor of the testis	1	1	100.0	0.0	0.0	0.0
	Yolk sac tumor	50	44	100.0	0.0	0.0	0.0
	Teratoma	50	41	95.1	0.0	0.0	4.9
	Squamous cell carcinoma of the penis	80	78	100.0	0.0	0.0	0.0

Tumors of endocrine organs (*n* = 1,171)	Adenoma of the thyroid gland	114	112	92.9	4.5	2.7	0.0
	Papillary thyroid carcinoma	392	379	89.4	9.0	1.1	0.5
	Follicular thyroid carcinoma	154	151	88.7	5.3	3.3	2.6
	Medullary thyroid carcinoma	111	108	79.6	16.7	1.9	1.9
	Parathyroid gland adenoma	43	42	100.0	0.0	0.0	0.0
	Anaplastic thyroid carcinoma	45	43	97.7	2.3	0.0	0.0
	Adrenal cortical adenoma	50	44	95.5	2.3	0.0	2.3
	Adrenal cortical carcinoma	26	26	88.5	11.5	0.0	0.0
	Phaeochromocytoma	50	49	100.0	0.0	0.0	0.0
	Appendix, neuroendocrine tumor (NET)	22	17	94.1	0.0	5.9	0.0
	Colorectal, neuroendocrine tumor (NET)	12	11	100.0	0.0	0.0	0.0
	Ileum, neuroendocrine tumor (NET)	49	49	100.0	0.0	0.0	0.0
	Lung, neuroendocrine tumor (NET)	19	18	100.0	0.0	0.0	0.0
	Pancreas, neuroendocrine tumor (NET)	97	94	45.7	17.0	9.6	27.7
	Colorectal, neuroendocrine carcinoma (NEC)	12	10	100.0	0.0	0.0	0.0
	Gallbladder, neuroendocrine carcinoma (NEC)	4	4	100.0	0.0	0.0	0.0
	Pancreas, neuroendocrine carcinoma (NEC)	14	14	64.3	7.1	14.3	14.3

Tumors of haematopoetic and lymphoid tissues (*n* = 353)	Hodgkin lymphoma	58	53	100.0	0.0	0.0	0.0
	Small lymphocytic lymphoma, B-cell type (B-SLL/B-CLL)	50	44	100.0	0.0	0.0	0.0
	Diffuse large B cell lymphoma (DLBCL)	113	103	100.0	0.0	0.0	0.0
	Follicular lymphoma	88	80	100.0	0.0	0.0	0.0
	T-cell non-Hodgkin lymphoma	25	24	100.0	0.0	0.0	0.0
	Mantle cell lymphoma	18	17	100.0	0.0	0.0	0.0
	Marginal zone lymphoma	16	14	100.0	0.0	0.0	0.0
	Diffuse large B-cell lymphoma (DLBCL) in the testis	16	16	100.0	0.0	0.0	0.0
	Burkitt lymphoma	5	2	100.0	0.0	0.0	0.0

Tumors of soft tissue and bone (*n* = 914)	Tenosynovial giant cell tumor	45	45	100.0	0.0	0.0	0.0
	Granular cell tumor	53	45	100.0	0.0	0.0	0.0
	Leiomyosarcoma	38	37	89.2	2.7	0.0	8.1
	Liposarcoma	132	130	100.0	0.0	0.0	0.0
	Malignant peripheral nerve sheath tumor (MPNST)	13	13	100.0	0.0	0.0	0.0
	Myofibrosarcoma	26	26	100.0	0.0	0.0	0.0
	Angiosarcoma	73	66	100.0	0.0	0.0	0.0
	Angiomyolipoma	91	88	87.5	9.1	0.0	3.4
	Dermatofibrosarcoma protuberans	21	17	100.0	0.0	0.0	0.0
	Ganglioneuroma	14	14	100.0	0.0	0.0	0.0
	Kaposi sarcoma	8	5	100.0	0.0	0.0	0.0
	Neurofibroma	117	104	95.2	4.8	0.0	0.0
	Sarcoma, not otherwise specified (NOS)	74	70	98.6	0.0	0.0	1.4
	Paraganglioma	41	41	100.0	0.0	0.0	0.0
	Ewing sarcoma	23	18	100.0	0.0	0.0	0.0
	Rhabdomyosarcoma	6	6	100.0	0.0	0.0	0.0
	Schwannoma	121	112	100.0	0.0	0.0	0.0
	Synovial sarcoma	12	11	100.0	0.0	0.0	0.0
	Osteosarcoma	43	39	100.0	0.0	0.0	0.0
	Chondrosarcoma	38	22	100.0	0.0	0.0	0.0
	Rhabdoid tumor	5	5	100.0	0.0	0.0	0.0

**Table 2:** Ranking of PR immunostaining in tumors (only tumor entities with ≥3 evaluable tumors were included in the ranking. Mammary tumors are italicized. Gynecological tumors are in bold).

Ranking PR	≥ weak (%)	≥ mod (%)	Strong (%)
*Tubular carcinoma of the breast*	82.6	73.9	65.2
**Granulosa cell tumor of the ovary**	81.1	51.4	21.6
*Mucinous carcinoma of the breast*	76.5	68.6	60.8
*Phyllodes tumor of the breast*	73.0	73.0	54.1
**Sertoli Leydig cell tumor of the ovary**	66.7	0.0	0.0
**Endometrioid endometrial carcinoma**	66.5	44.7	31.0
**Endometrioid carcinoma of the ovary**	62.0	43.5	29.3
*Invasive breast carcinoma of no special type*	57.1	46.5	35.8
*Lobular carcinoma of the breast*	57.0	47.0	37.4
Pancreas, neuroendocrine tumor (NET)	54.3	37.2	27.7
**Leydig cell tumor of the ovary**	50.0	0.0	0.0
Leydig cell tumor of the testis	36.7	3.3	0.0
Pancreas, neuroendocrine carcinoma (NEC)	35.7	28.6	14.3
**Carcinosarcoma of the ovary**	34.1	11.4	4.5
**Serous carcinoma of the ovary**	32.1	10.0	5.8
**Endometrial serous carcinoma**	20.6	5.9	2.9
Medullary thyroid carcinoma	20.4	3.7	1.9
**Endometrial carcinoma, high grade, G3**	16.7	8.3	8.3
Small cell neuroendocrine carcinoma of the prostate	16.7	0.0	0.0
Small cell carcinoma of the lung	12.5	6.3	0.0
Angiomyolipoma	12.5	3.4	3.4
**Carcinosarcoma of the uterus**	12.2	7.3	7.3
*Medullary carcinoma of the breast*	12.1	9.1	6.1
Adrenal cortical carcinoma	11.5	0.0	0.0
Follicular thyroid carcinoma	11.3	6.0	2.6
**Clear cell carcinoma of the ovary**	11.1	4.4	2.2
Leiomyosarcoma	10.8	8.1	8.1
Papillary thyroid carcinoma	10.6	1.6	0.5
**Adenocarcinoma of the cervix**	9.5	4.8	0.0
Adenoma of the thyroid gland	7.1	2.7	0.0
Oncocytoma	6.9	0.4	0.0
Myoepithelial carcinoma of the salivary gland	5.9	5.9	0.0
Appendix, neuroendocrine tumor (NET)	5.9	5.9	0.0
**Mucinous carcinoma of the ovary**	5.2	5.2	5.2
**Brenner tumor**	5.0	5.0	5.0
Teratoma	4.9	4.9	4.9
Neurofibroma	4.8	0.0	0.0
Chromophobe renal cell carcinoma	4.6	1.3	1.3
Adrenal cortical adenoma	4.5	2.3	2.3
Small cell neuroendocrine carcinoma of the bladder	4.3	0.0	0.0
Clear cell (tubulo) papillary renal cell carcinoma	4.2	4.2	0.0
Adenocarcinoma NOS of the salivary gland	2.5	2.5	1.2
Anaplastic thyroid carcinoma	2.3	0.0	0.0
Ductal adenocarcinoma of the pancreas	2.2	1.2	0.6
Urothelial carcinoma of the kidney pelvis	1.6	0.0	0.0
Sarcoma, not otherwise specified (NOS)	1.4	1.4	1.4
**Squamous cell carcinoma of the cervix**	0.8	0.0	0.0
Adenocarcinoma of the prostate (recurrence)	0.4	0.0	0.0
Urothelial carcinoma, pT2-4 G3	0.3	0.3	0.3
Mucoepidermoid carcinoma of the salivary gland	0.3	0.3	0.0
Papillary renal cell carcinoma	0.3	0.0	0.0
Adenocarcinoma of the colon	0.1	0.0	0.0
Clear cell renal cell carcinoma	0.1	0.0	0.0

**Table 3:** PR immunostaining and tumor phenotype in breast carcinoma of no special type, endometrioid endometrial carcinoma, high-grade serous ovarian carcinoma, and pancreatic neuroendocrine tumors.

			*n*	Progesterone receptor IHC result				*p*
				Negative (%)	Weak (%)	Moderate (%)	Strong (%)	
Breast carcinoma of no special type	Tumor stage	pT1	749	36.7	9.9	11.9	41.5	<0.0001
		pT2	613	44.5	11.4	11.4	32.6	
		pT3-4	122	54.9	11.5	4.1	29.5	
	Grade	G1	183	23.0	10.9	12.6	53.6	<0.0001
		G2	799	34.3	10.4	13.4	41.9	
		G3	543	59.7	10.7	7.2	22.5	
	Nodal stage	pN0	682	40.2	8.8	9.8	41.2	<0.0001
		pN1	325	38.2	14.5	14.2	33.2	
		pN2	114	47.4	13.2	10.5	28.9	
		pN3	68	63.2	13.2	5.9	17.6	
	Distant metastasis	pM0	199	38.7	7.5	10.6	43.2	<0.0001
		pM1	104	64.4	11.5	5.8	18.3	
	HER2 status	Negative	850	37.8	10.5	11.1	40.7	<0.0001
		Positive	120	63.3	12.5	6.7	17.5	

Endometrioid endometrial carcinoma	Tumor stage	pT1	94	29.8	22.3	13.8	34.0	0.8885
		pT2	23	34.8	17.4	8.7	39.1	
		pT3-4	29	34.5	24.1	17.2	24.1	
	Nodal stage	pN0	43	20.9	25.6	18.6	34.9	0.0327
		pN+	25	56.0	16.0	8.0	20.0	

Serous ovarian carcinoma	Tumor stage	pT1	33	51.5	18.2	9.1	21.2	0.0750
		pT2	43	69.8	20.9	2.3	7.0	
		pT3	267	72.3	18.7	3.7	5.2	
	Nodal stage	pN0	83	65.1	26.5	6.0	2.4	0.0534
		pN1	171	76.0	15.8	2.3	5.8	

Pancreatic neuroendocrine tumors	Tumor stage	pT1	10	40.0	10.0	20.0	30.0	0.0954
		pT2	15	26.7	33.3	6.7	33.3	
		pT3	22	68.2	4.5	13.6	13.6	
		pT4	2	0.0	50.0	0.0	50.0	
	Nodal stage	pN0	24	29.2	20.8	16.7	33.3	0.0345
		pN+	21	71.4	9.5	9.5	9.5	

## Data Availability

All data generated or analyzed during this study are included in this published article.

## Supplementary Materials

Supplementary 1 Supplementary Figure 1: (A–D) PR staining with antibody MSVA-570R. (E–H) PR staining with antibody PgR636. Absent staining in mucosa of the ileum (A) and thyroid gland (B) with MSVA-570R and moderate to strong staining with PgR636 in goblet cells in the mucosa of the ileum and in colloid of the thyroid gland. Identical staining of both antibodies in islets of Langerhans in the pancreas (C and G) and endometrium (D and H).

Supplementary 2 Supplementary Figure 2: PR positivity and overall survival in patients with invasive breast carcinomas of no special type.

Supplementary 3 Supplementary Figure 3: graphical representation of PR data from this study (marked with a cross) in comparison with the previous literature (marked with a dot). In order to simplify the figure the percentage of weak, moderate and strong staining was merged. Red dots are used for studies from previous studies involving 3-10 cases, yellow dots for studies involving 11-25 cases, and green dots for studies involving >25cases. All studies are quoted in the supplementary material.
