# Supplementary figures and images for: Diagnostic and Prognostic Impact of Progesterone Receptor Immunohistochemistry: A Study Evaluating More Than 16,000 Tumors
Source: Anal Cell Pathol (Amst). 2022 Aug 8;2022:6412148. doi: 10.1155/2022/6412148 (PMC9381849; doi:10.1155/2022/6412148)

Antibody:  
MSVA-570R

A

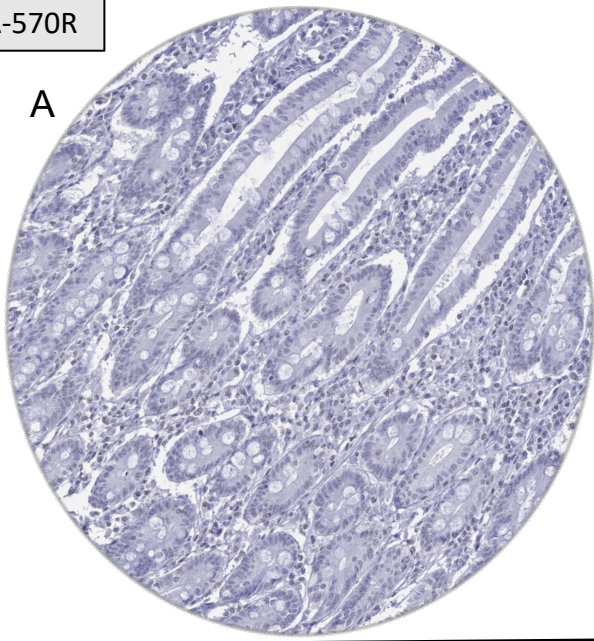

B

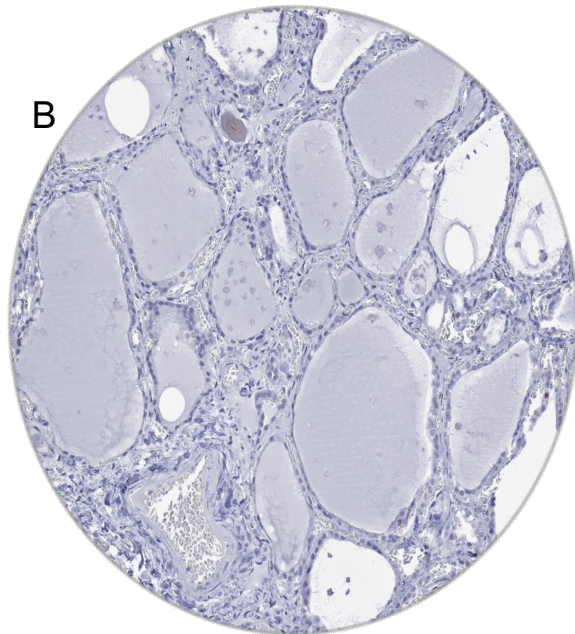

C

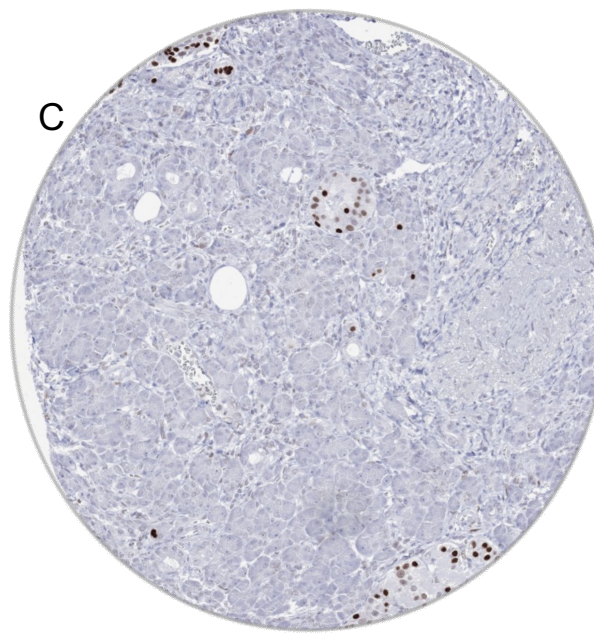

D

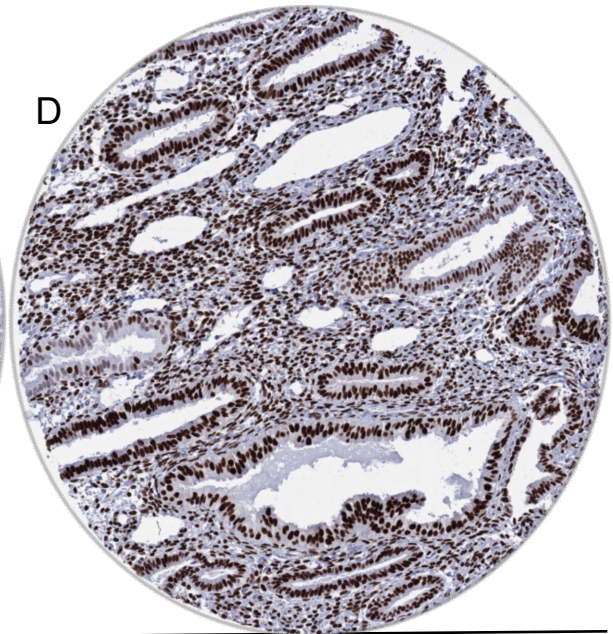

Antibody:  
PgR636

E

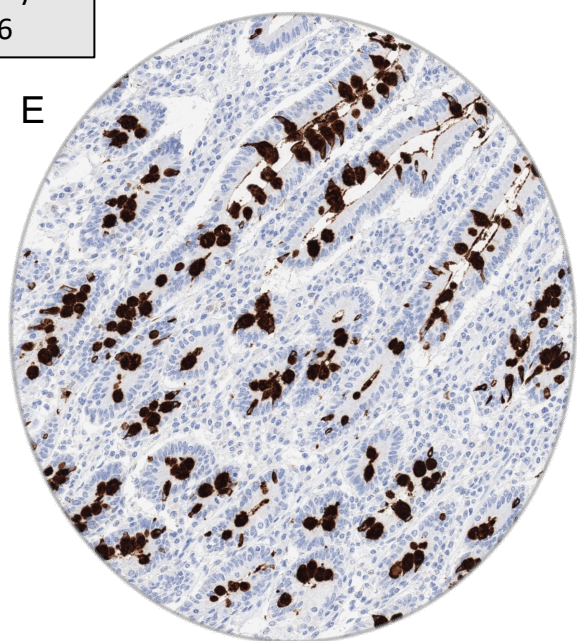

F

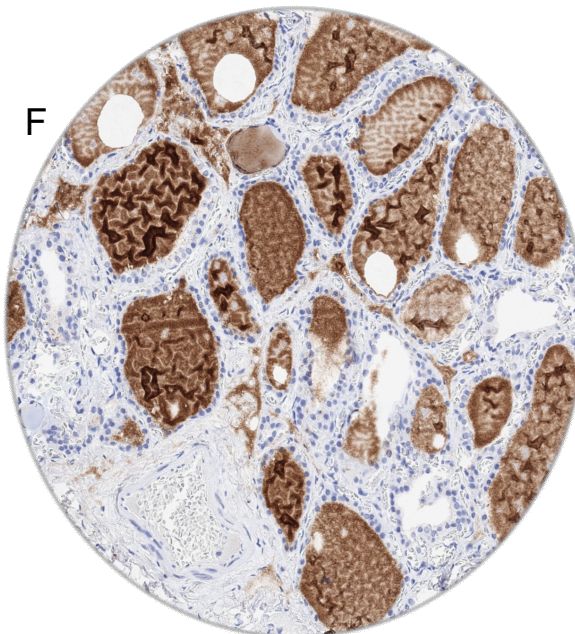

G

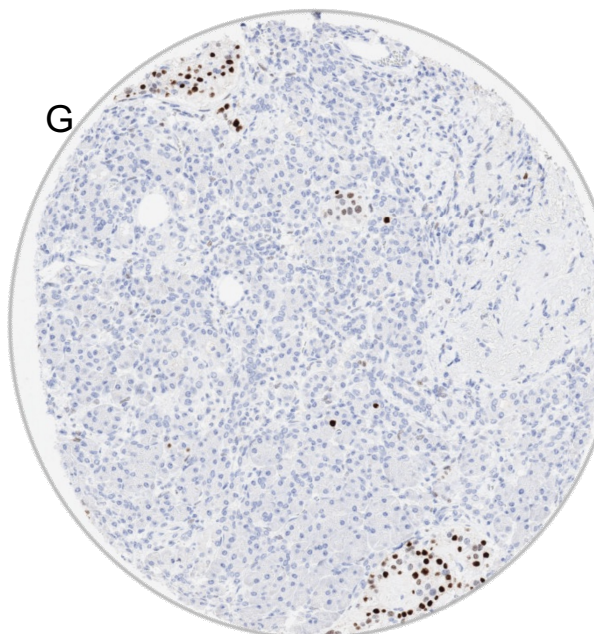

H

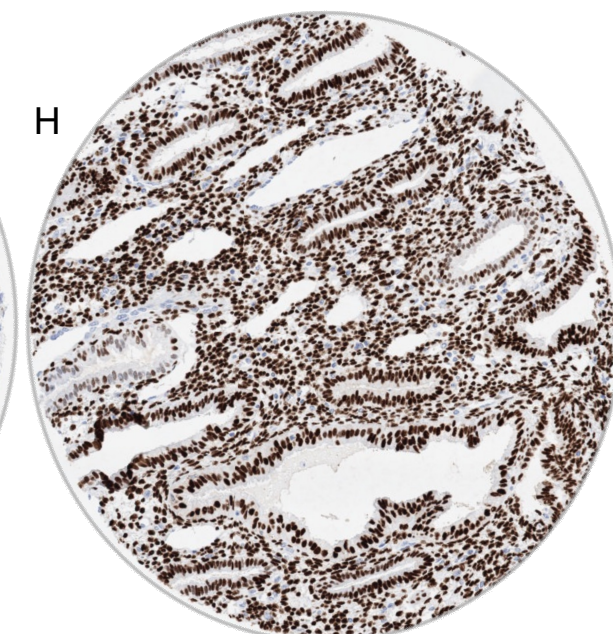

Supplement: Supplementary 1 — Supplementary Figure 1: (A–D) PR staining with antibody MSVA-570R. (E–H) PR staining with antibody PgR636. Absent staining in mucosa of the ileum (A) and thyroid gland (B) with MSVA-570R and moderate to strong staining with PgR636 in goblet cells in the mucosa of the ileum and in colloid of the thyroid gland. Identical staining of both antibodies in islets of Langerhans in the pancreas (C and G) and endometrium (D and H). [file 6412148.f1.pdf]

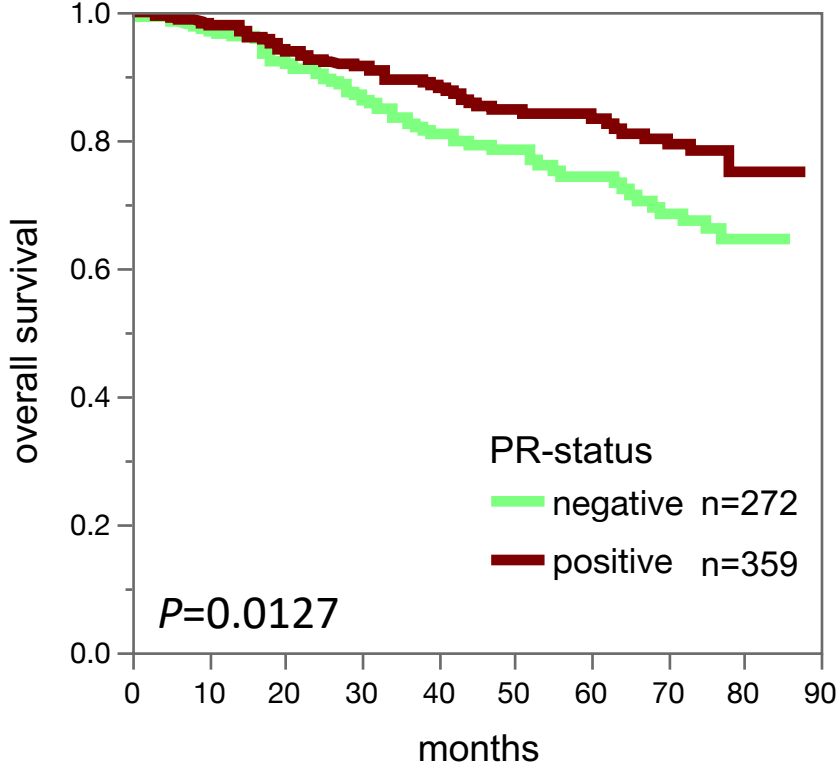

Supplement: Supplementary 2 — Supplementary Figure 2: PR positivity and overall survival in patients with invasive breast carcinomas of no special type. [file 6412148.f2.pdf]

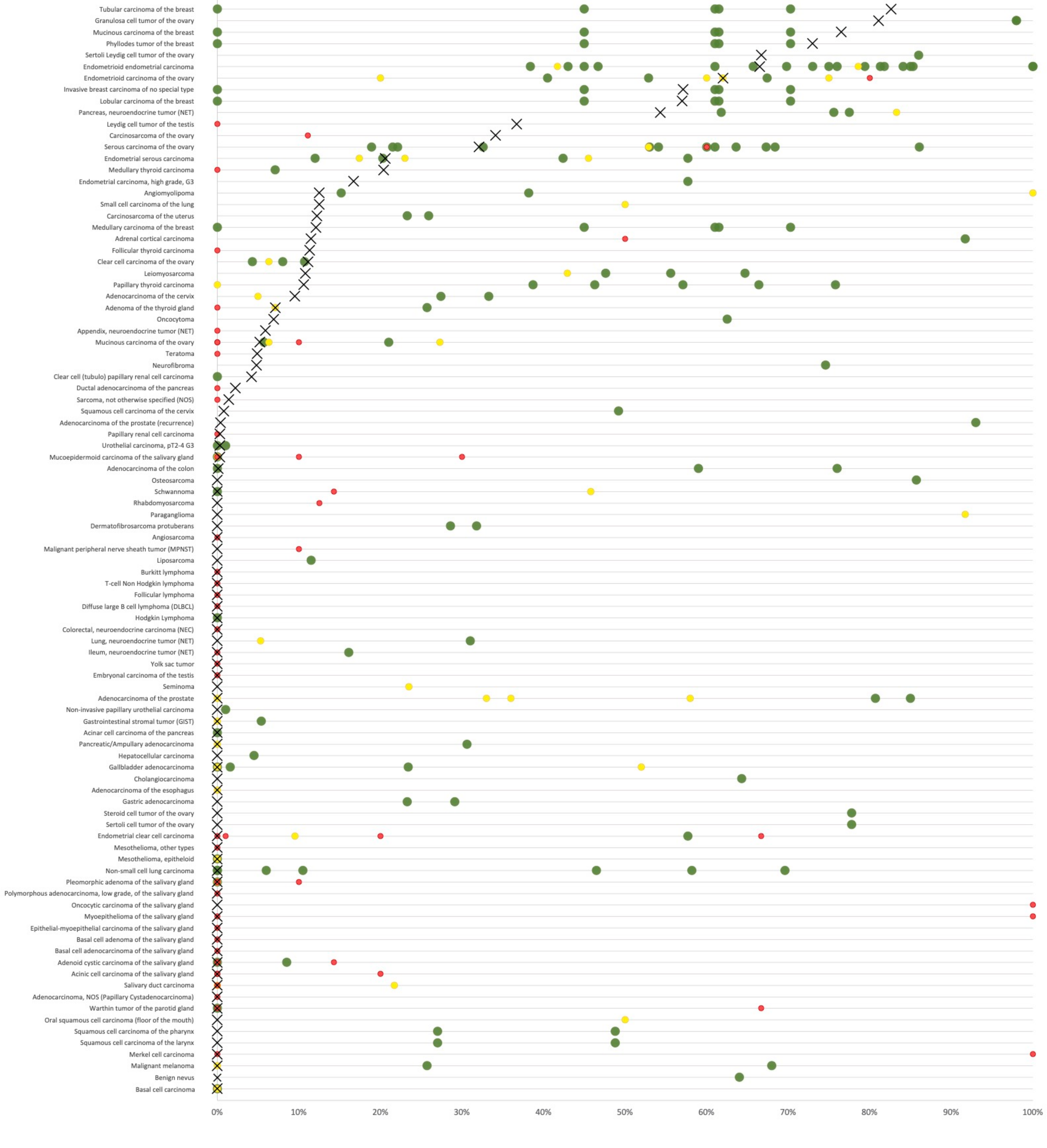

Supplement: Supplementary 3 — Supplementary Figure 3: graphical representation of PR data from this study (marked with a cross) in comparison with the previous literature (marked with a dot). In order to simplify the figure the percentage of weak, moderate and strong staining was merged. Red dots are used for studies from previous studies involving 3-10 cases, yellow dots for studies involving 11-25 cases, and green dots for studies involving >25cases. All studies are quoted in the supplementary material. [file 6412148.f3.pdf]
